# Recent increase in pertussis incidence in Korea: an age-period-cohort analysis

**DOI:** 10.4178/epih.e2021053

**Published:** 2021-08-18

**Authors:** Chanhee Kim, Seonju Yi, Sung-il Cho

**Affiliations:** 1 Department of Disease Control Policy, Gyeonggi Provincial Government, Suwon, Korea; 2 Central Disease Control Headquarters, Korea Disease Control and Prevention Agency, Cheongu, Korea; 3 Graduate School of Public Health, Seoul National University, Seoul, Korea

**Keywords:** Vaccines, Immunization, Whooping cough, Pertussis, Age-period-cohort analysis

## Abstract

**OBJECTIVES:**

Pertussis or whooping cough—one of the most contagious diseases—is caused by the Gram-negative bacterium *Bordetella pertussis*. Despite a high vaccination rate, Korea recently experienced a resurgence of pertussis. This study explores patterns and possible explanations for this resurgence through an age-period-cohort analysis.

**METHODS:**

Using secondary data from the infectious disease portal of the Korea Disease Control and Prevention Agency and the Korea Statistical Information Service of Statistics Korea, this study analyzed the incidence of pertussis in Korea to determine which factors contributed to the recent increase using an age-period-cohort model.

**RESULTS:**

Analysis of the age effect indicated that the age group most vulnerable to pertussis was 0-year to 2-year-olds. Analysis of the period effect showed a sharp increase in the incidence rate after 2016. Analysis of the cohort effect showed a significant decrease in incidence beginning with the 1955 birth cohort, with the risk increasing again with the 2000s birth cohort.

**CONCLUSIONS:**

Previous studies have suggested 3 main possible explanations for our results. First, the increased incidence rate can be attributed to contact rates. Second, the rate of immunity through natural exposure has decreased due to the low number of circulating pathogens, in turn affecting the trend of infection. Lastly, variations in pathogens may have also contributed to the increase in incidence. Given that the most significant increase in incidence was observed among infants younger than 1 year old, sufficient maternal immunity must be prioritized to provide passive immunity to newborns via the placenta.

## INTRODUCTION

Pertussis, which is one of the most contagious known diseases, is caused by the Gram-negative bacterium *Bordetella pertussis* [1]. *B. pertussis* infection damages the ciliated respiratory epithelium and can lead to functional failure and inflammation of the respiratory tract [2,3]. Another name for pertussis, whooping cough, originated from its distinguishing symptom in which a “whoop” sound is generated by patients during inhalation due to the narrowed airways [1,2,4]. Humans are the only known host of *B. pertussis* [1]. Pertussis is typically developed by young children, but anyone of any age can be affected [4,5]. Infants, however, are the population most vulnerable to pertussis due to their immature respiratory systems and incomplete vaccination schedules [4]. While adolescents and adults tend to only experience relatively mild symptoms [5], they can be a vector of pertussis to vulnerable populations, including infants and children who are not fully immunized [6].

According to the World Health Organization, the number of pertussis cases and deaths has decreased significantly due to the high rate of diphtheria-tetanus-pertussis (DTaP) vaccination coverage since the 1980s [7]. While the global burden of pertussis has decreased substantially, the burden of pertussis on a national level varies across countries. In low-income countries, it is difficult to obtain an exact estimate of the disease burden due to poor surveillance systems and a lack of diagnostic tools [8]. However, even with these constraints, pertussis is still estimated to be one of the most fatal diseases affecting infants who are 1 year old or younger in developing countries, who make up 90% of the disease burden of pertussis worldwide [9].

Resurgences in pertussis cases have been reported since the 1980s in developed countries, including Canada, the United States, Australia, and across Europe, despite high rates of vaccination coverage [10]. This recent pattern of pertussis outbreaks has a cycle ranging from 2.0 years to 4.6 years [11]. There have been few increases in the rate of pertussis among adolescents and adults in the past [11], and the recent increase in the incidence of pertussis among adolescents and adults may pose a threat to young children who have incomplete immunity against pertussis whose parents or siblings may have been exposed to pathogens [12]. Korea has also experienced a resurgence of pertussis beginning in the 2000s, similar to that of other highly vaccinated countries, including outbreaks in schools and postpartum centers [2,13-15]. The outbreak pattern is similar to that of other highly vaccinated countries, with a cycle of 2-3 years [2].

Many studies have examined various possible reasons for the resurgence of pertussis, including the relatively short duration of immunity of the DTaP vaccine [4,16], genetic changes in circulating pathogens [17-19], and the lower likelihood of developing natural immunity due to a decrease in the incidence of pertussis [20]. However, the resurgence of the disease cannot be explained by any single factor. Analysis of the age, period, and cohort effects may provide insight into the mixed effects of various factors. In this study, we compared the results of an age-period-cohort (APC) analysis with the results of preceding studies to better identify the causes of the recent re-emergence of pertussis in Korea and provide a basis for the development of policies related to pertussis prevention.

## MATERIALS AND METHODS

First, pertussis cases and mid-year population data were collected to calculate the annual age-specific incidence of pertussis. In Korea, physicians are required to report all cases of pertussis once identified, including both laboratory-confirmed and clinically suspected cases. Aggregated data related to diseases that require physician reporting were obtained from the infectious disease portal of the Korea Disease Control and Prevention Agency (KDCA, http://www.kdca.go.kr/npt). This study used age-specific pertussis data from 2001 to 2018. In addition, age-specific mid-year population data were obtained from the Korea Statistical Information Service of Statistics (http://www.kosis.kr).

Using open data from the infectious disease portal, trends in the incidence of pertussis were analyzed by age, year, and case type. The KDCA classifies reported cases as either confirmed or suspected cases [21]. A confirmed case is defined as a patient who presents pertussis-compatible clinical symptoms for whom a positive laboratory test result is obtained. Suspected cases are defined as patients whose clinical symptoms and epidemiological relationship suggest pertussis, but for whom laboratory test results are unavailable [21]. For annual age-specific data, the open data source used in this study provided the total number of cases without stratifying by case type (confirmed or suspected). We used the total number of cases for our main analysis and examined the annual trend by case type without considering age information.

Korea began administering the diphtheria-tetanus-whole cell pertussis (DTwP) vaccination in 1955, which was changed to the DTaP vaccine in 1989 due to safety issues. Thus, people who were born before 1955 (the pre-1955 cohort) have never received any artificial immunity, people born between 1955 and 1988 received the DTwP vaccine, and people born after 1988 received the DTaP vaccine.

Before the APC analysis, we determined the annual age-specific incidence of pertussis per age group using data from the infectious disease portal (pertussis cases) and Korea Statistics (mid-year population). Age was divided into 7 groups: infants (0 years old), toddlers (1-3 years old), preschoolers (4-6 years old), school-aged children (7-11 years old), adolescents (12-19 years old), adults (20-59 years old), young seniors (60-74 years old), and old seniors (≥ 75 years old). These groupings were used to identify different patterns of change between people of different ages.

Goodness-of-fit statistics were based on the assumption that age, period, and cohort effects all occurred and needed to be estimated simultaneously [22]. The most appropriate model was identified by evaluating the model fit statistics with possible sets and combinations of coefficients age (A), period (P), and cohort (C): AP, AC, PC, and APC [22]. A deviance value for a model that is close to the degree of freedom indicates that the model is well-fitted, whereas a large deviance indicates that the model is a poor fit [23].

APC analysis is a type of multiple regression comprised of 3 time-varying components: age, period, and cohort [24]. It is usually used to identify a possible hypothesis related to specific health problems. Prior studies used APC analysis to examine the recent increase in the burden of scarlet fever and varicella in Korea [25,26]. APC analysis is also applicable for examining non-communicable diseases. One well-known study that used APC analysis was able to identify the cause of the sharp increase in thyroid cancer incidence in Korea as being related to a recent increase in thyroid screening [27].

The age effect refers to the consequences of aging, including a combination of exposure to risks and physiological changes to the body [22,24]. The period effect refers to historical circumstances, an individual’s economic status at the time of infection, and the availability of new medical techniques [24]. The previous study on the increase in thyroid cancer incidence in Korea identified the period effect as expanded screening for thyroid cancer [27]. The cohort effect refers to shared outcomes among specific populations born in the same year [28]. For example, Choe et al. [29] found that the cohort that was vaccinated for mumps with the ineffective Rubini strain likely contributed to a resurgence in mumps. Further information on the methodology is available in the Supplementary Material 1.

The Poisson APC model was adopted to estimate the age, period, and cohort effects on the trend of pertussis incidence from 2001 to 2018 in Korea. The formula for the model is as follows: ln [λ(a, p)]= f(a)+g(p)+h(c), where a, p, and c refer to the effect of age, period, and cohort, respectively.

However, because of the linear-dependent relationship between the 3 variables (cohort= period−age), the APC model fails to meet the basic assumption of multiple regression that each variable is independent of the others, which is referred to as an “identification problem” [24]. To overcome the identification problem arising from the linear dependence of the 3 factors, the intrinsic estimator (IE) method was applied [24]. The IE method is less subjective than the traditional constraining model (constrained general linear model) in which a researcher chooses 1 of the 3 variables to constrain [30].

Plotting of the incidence by age group, as described earlier, is useful for examining the overall trend of a disease, but equally spaced blocks must be set for APC analysis. Blocks were set using 3 separate units: age, calendar year, and birth cohort. Patients older than 80 were excluded due to their low incidence of pertussis (67 of 2,268 cases, or 0.03%). There were 27 age groups in total (ranging from those aged 0-2 to 78-80), 18 calendar years from 2001 to 2018, and 32 birth cohorts from 1923-1925 to 2016-2018. However, after excluding data on individuals who were over 80 years old, there were several cells with missing values. To analyze data with a sufficient sample size, the lowest incidence was replaced with a value of 0 [31]. All statistical analyses were performed using Stata version 13 (StataCorp., College Station, TX, USA).

### Ethics statement

The Institutional Review Board of Seoul National University waived the need for an ethics review given that all data were publicly available and lacked any personal identifiers.

## RESULTS

**Figure 1.**
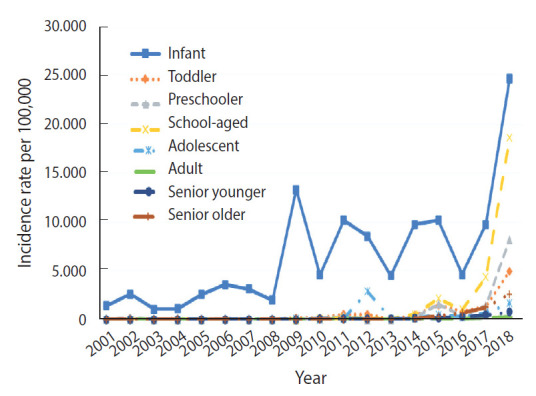
Incidence of pertussis by age group, Korea, 2001-2018.

Figure 1 shows the secular trend of pertussis incidence from 2001 to 2018 in Korea. The incidence increased across all 8 groups after 2017, and 2018 had the highest incidence rate. Infants (0 years old) had the highest incidence rate and the highest increase in incidence from 2017 to 2018, followed by school-aged children (7-11 years old). Table 1, which illustrates the incidence of pertussis by age and calendar year, shows an increase of 17.6 times in pertussis cases among infants in 2018 since 2001. In addition, there was an increase in pertussis cases among school-aged children by 93.5 times in 2018 since 2011, when the incidence among this age group was close to zero. This was the most explosive increase among all age groups, which is similar to the trend of pertussis in many other highly vaccinated countries.

**Figure 2.**
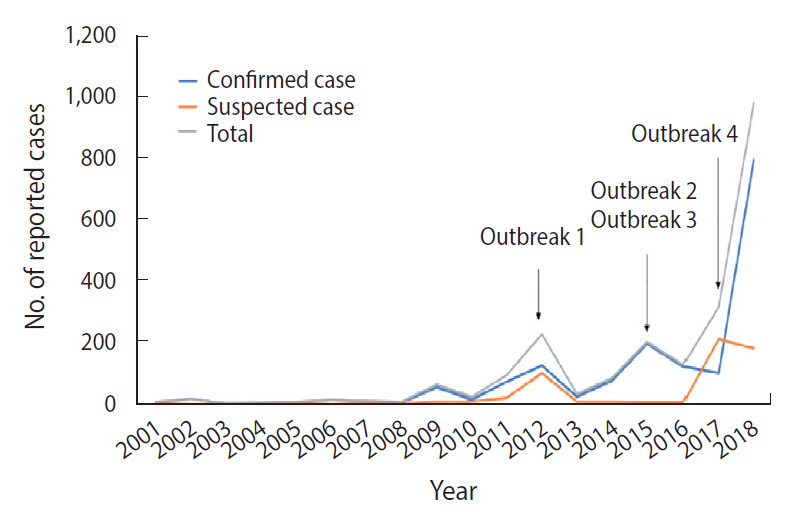
Number of classification-specific reported cases by year, Korea, 2001-2018.

The secular trend of pertussis by case type and outbreak occurrence are shown in Figure 2. Outbreaks 1-4 refer to the reported outbreaks of pertussis in Korea. Outbreak 1, which occurred in Yeongam-gun in 2012, occurred in middle and high school dormitories [2,14]. Outbreaks 2 and 3 occurred in 2015 at postpartum care centers in Andong-si and elementary schools in Changwon-si, respectively [2,15]. The last outbreak, reported in 2017, occurred in Gwangju-si in Gyeonggi-do and Sejong-si [2,13]. All cases were confirmed via laboratory tests for outbreaks 2 and 3, whereas suspected cases were also included during the investigation of the other outbreaks.

The result of the goodness-of-fit test suggested that the full APC (IE) model had the best fit for the APC analysis (Table 2). The residual deviance represents the goodness-of-fit of the models. According to the residual deviance, the full APC model, which showed the smallest residual deviance, most appropriately represented the data.

**Figure 3.**
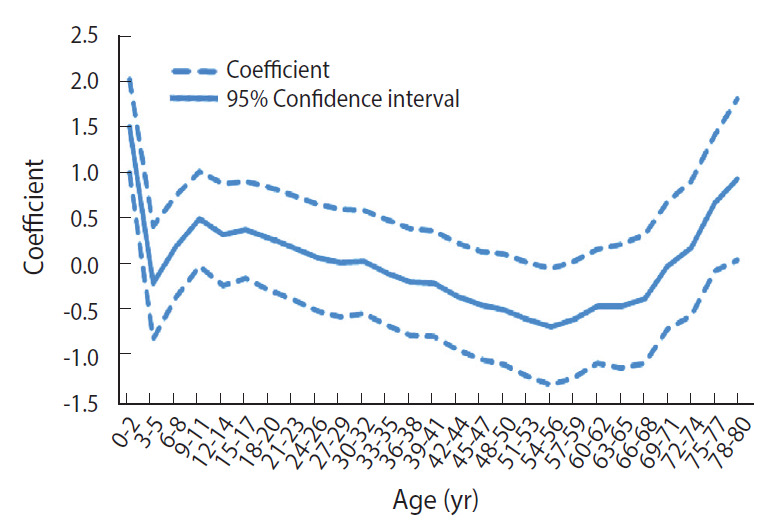
Intrinsic estimator (coefficient) of age effect from age-specific incidence (100,000 person) of pertussis by age groups.

The effects of age on the incidence of pertussis are shown in Figure 3. The results indicate that the most vulnerable age group to pertussis was 0-year to 2-year-olds, which includes infants who received the initial 3 doses of the DTaP vaccination. A sharp decrease was observed in the risk of pertussis among 3-year to 5-year-olds following the third vaccination. However, the risk increased again among 9-year to 11-year-olds and did not decrease again until the 54-year to 56-year-old group. After the lowest incidence rate—observed in 54-year to 56-year-olds—the risk steadily increased with each age group.

**Figure 4.**
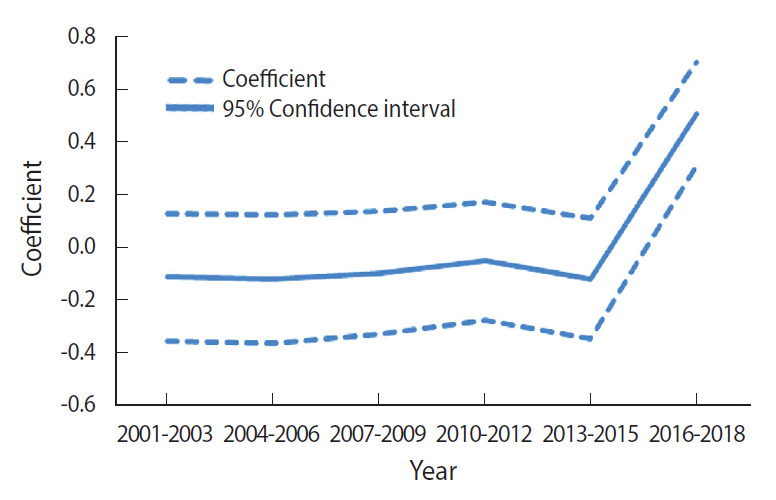
Intrinsic estimator (coefficient) of period effect from age-specific incidence (100,000 person) of pertussis by calendar years.

The period effect was also significant, as shown in Figure 4. From 2001 to 2015, there were no significant changes in the risk of pertussis. Beginning in 2016, the risk of pertussis sharply increased, showing a similar trend in the overall incidence of pertussis inh Korea from 2001 to 2018.

**Figure 5.**
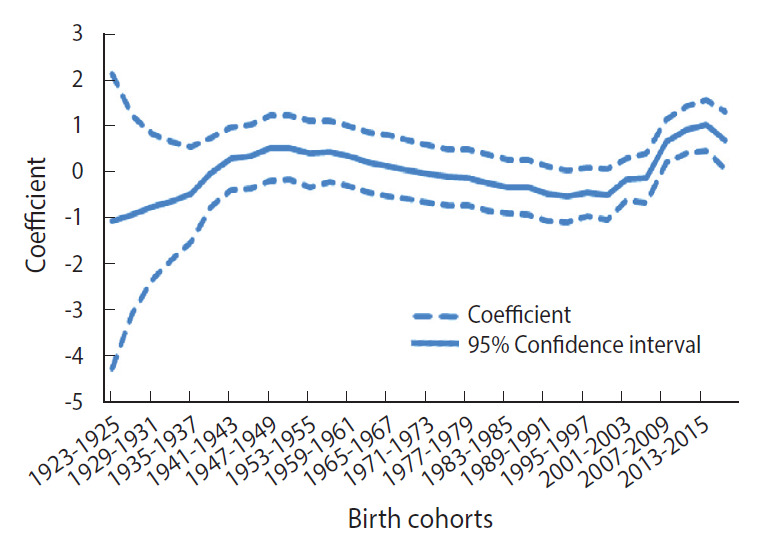
Intrinsic estimator (coefficient) of cohort effect from age-specific incidence (100,000 person) of pertussis by birth cohorts.

Another increase in the risk of pertussis was observed related to the cohort effect (Figure 5). The risk of pertussis increased gradually until the introduction of vaccination in 1955. After that, there was a steady decrease in the risk of pertussis until the early 1990s birth cohorts. Beginning with birth cohorts in the 2000s, the risk of pertussis began to increase again, becoming as high as it was before the vaccine was introduced.

## DISCUSSION

We described the secular trend of pertussis incidence and estimated the effects of age, period, and cohort from 2001 to 2018 in Korea. The most significant increases were observed among infants, school-aged children, and preschoolers. The exceptionally high proportion of adolescents who contracted pertussis in 2012 seemed to have resulted from pertussis outbreaks that took place in middle and high school dormitories. From 2001 to 2018, the average reported number of pertussis cases among adolescents was 17.1 cases per 100,000 people. However, in 2012, a total of 157 cases were reported, of which 113 were from a high school and 41 from a middle school in Yeongam-gun [14].

Given that the routine vaccination schedule of the DTaP vaccine includes a 5-dose series administered at 2 months, 4 months, 6 months, 15-18 months, and 4-6 years, age had a preventive effect after the first 5 vaccine doses, which are completed at the age of 4-6 years. Despite the vaccine’s effectiveness, the risk of pertussis increased soon after completion of the vaccination cycle among children aged 6-8 and 9-11. This suggests a scientific rationale for widely administering the tetanus-diphtheria-pertussis (Tdap) vaccine, which is recommended for 11-year to 12-year-olds. We can infer that increases in the incidence rate of pertussis among school-aged children occurred due to the increased rate of social contact in the school environment. According to a study on the age-specific contact rate of respiratory diseases, the highest contact rate was observed among 10-year to 14-year-olds [32]. Studies supporting this contact rate pattern have been conducted in Japan and the Netherlands. In Japan, elementary school children have been found to be more vulnerable to pertussis than junior high school students due to their lower exposure rate to pertussis [33]. Similarly, in the Netherlands, children who completed their 5-dose vaccination series were found to already have sufficiently high-level immunity at 9 years old owing to natural immunity boosting [34].

The age-specific contact rate also explains the decreasing pattern that occurred among those slightly older than 9-year to 11-year-olds, in which age-specific contact rates began to decrease at the age range of 15-19 [32]. In addition, in Korea, the School Entry Requirement of Certification Program conducted by the KDCA requires Tdap immunization certification both in elementary school and in middle school. This boost in the vaccination rate likely had an effect on the decrease in risk after the ages of 9-11.

From the results of our analysis on the effects of age, we can conclude that, despite a lower incidence rate of pertussis, there were still circulating pathogens of pertussis to stimulate immunity-boosting reactions to the disease. This effect may increase with age, resulting in a decreasing trend of risk until middle age. The steadily increasing trend that was observed beginning at the age of 60 indicates that individuals develop a lower immunity to the disease with age [35].

The period effect on the risk of pertussis was also significant, with the sharpest increase in risk occurring from 2016 to 2018. Based on overall incidence (Figure 1), the most significant increase in pertussis occurred in 2018, and the results of the analysis on the period effect reflect this pattern. Three possible explanations have been suggested in previous studies as an explanation for this finding. First, it has been proposed that incidence rates increased due to antigenic variants of pertussis that do not correspond to the different vaccine types [36]. In Korea, the type of pathogen circulating in 2011 and 2012 was found to be different from the one that circulated from 2000 to 2009 [36]. The same study also revealed that notable changes in *B. pertussis* genotypes in Korea have emerged, especially concerning genes that determine antigenicity, as has already been reported in many other countries [36].

Second, other studies have suggested that there are fewer opportunities to boost natural immunity due to the reduced number of circulating pathogens [20]. Because of the reduced scale of infections due to the lower incidence, the size of the population without any re-exposure to the pertussis pathogen has grown larger than it was in the past [20]. The increase in the size of this population beyond the threshold of protection is likely to have led to a rapid increase in the incidence of pertussis [37].

Lastly, the outbreak of Middle East respiratory syndrome (MERS) in 2015 increased healthcare providers’ awareness of respiratory diseases. Since the reporting criteria for pertussis included suspected cases, the sharp increase observed from 2013 to 2015 may have reflected changes in diagnostic and reporting patterns in relation to the 2015 outbreak in Korea. The effect of the MERS outbreak on reporting behavior has been detailed in a previous study in which the results of an interrupted time series analysis showed an increase in the overall reporting of notifiable diseases since the MERS outbreak [38].

According to the results of analysis on the cohort effect shown in Figure 5, the risk of pertussis increased until the introduction of the DTwP vaccine in 1955. Beginning with the 1955 birth cohort, the risk steadily decreased up to the late 1990s birth cohorts. The increase in risk beginning with the 2000s birth cohort may have been a result of reduced maternal passive immunity. During the pre-vaccine era, the most vulnerable population was not infants younger than 1 year old but rather toddlers aged 1-4 [39,40]. Moreover, during the pre-vaccine era, case fatality rates of pertussis were higher among 2-month and 3-month-old infants than among 1-month-old infants, thus showing indirect evidence of maternal immunity transfer to infants [41]. However, waning immunity after vaccination, accompanied by a decreased rate of circulating pathogens, makes it difficult for pregnant women to have sufficient antibodies against pertussis to transfer to their newborns [41]. Thus, the increased risk among the 2000s birth cohort may have resulted from the decreased immunity of mothers.

In summary, 3 significant possible reasons were suggested in previous studies that explain the results of the present study. First, contact rates correspond to increases and decreases in pertussis incidence. Thus, the pattern of the age effect can be explained via the age-specific contact rates. Exceptional patterns of incidence at both ends of the age range can be explained by the effects of vaccination during childhood and decreased immunity with age, respectively.

Another factor affecting the trend of pertussis is the decrease in opportunities for natural immunity boosting due to the low number of circulating pathogens. The introduction of vaccines resulted in decreased incidence, but it also increased the population of those susceptible to the disease due to the combined effect of waning immunity after vaccination and the absence of opportunities for natural immunity boosting. This finding is linked to a decrease in passive immunity from mothers, which is reflected in the analysis of the cohort effect. The mothers of those in the most recent birth cohort (2000s) were vaccinated during childhood and, in addition, had insufficient exposure to pertussis for natural immunity boosting to take place.

Lastly, variations in pathogens have also been suggested as an explanation for recent trends in the incidence of pertussis. This hypothesis is supported by a serological study conducted in Korea. It is assumed that the variation discovered in the serological study resulted from the adaptation of the pathogen to the vaccine.

From a public health perspective, providing more opportunities for immunity boosting is a necessary intervention for improving the current situation brought resulting from the aforementioned 3 factors that caused the recent increase in pertussis in Korea. Given that the most significant increase in incidence occurred among infants younger than 1 year old, it is crucial for mothers to develop sufficient immunity levels to provide their infants, who are too young to be vaccinated, with passive immunity via the placenta.

According to a randomized clinical trial conducted in the United States, no increased risk of adverse events were observed among women or their infants after receiving the Tdap vaccine during weeks 30-32 of pregnancy. Additionally, high concentrations of pertussis toxin antibodies were observed in infants during their first 2 months of life, and no altered response to the DTaP was observed [42]. Another randomized controlled trial conducted in the Netherlands also indicated that maternal immunity during pregnancy prevented neonates from contracting pertussis [43].

Although our study findings are consistent with those of previous studies, this study has a few limitations. First, given the relatively low reporting rates of vaccine-preventable diseases (VPDs) compared to those of other notifiable diseases, the entire disease burden of pertussis may have been underestimated in this study. According to a previous study that calculated the reporting and claim ratio (R/C ratio) of infectious diseases in Korea, no VPDs had an R/C ratio close to 1 or even exceeding 0.5 [38]. Second, the data did not provide information about vaccination history, meaning that the study results made an assumption regarding the effects of vaccination. In future studies, information about vaccination history may enable researchers to determine the effects of vaccination more precisely by calculating the attack rate of pertussis among the vaccinated population. Third, the fundamental limitation of APC analysis is that the tool cannot determine an exact association between the putative causal factors and the phenomenon. We can explain the phenomenon resulting from the APC analysis based on existing studies; however, no direct measurement of potential determinants was included in this study.

Lastly, due to the inclusion of suspected cases in the analysis, the study results should be interpreted carefully since some of the suspected cases may have been false positives. If certain age groups, especially the youngest or oldest age groups, had a higher proportion of suspected cases, the relative risk among these age groups could have been overestimated in the age effect curve.

Despite these limitations, this study provides epidemiological evidence concerning the patterns of pertussis in Korea. Further data on vaccination histories and age-specific data from previous periods would enable more accurate estimates.

In conclusion, this study examined the age, period, and cohort effects of pertussis in Korea. The standard vaccination schedule in Korea mainly focuses on children. However, this study, through APC analysis, suggests the importance of adult vaccination, especially among pregnant women. Korea is one of the countries with the best prenatal care globally. The importance of maternal vaccination must be emphasized and promoted in collaboration with local hospitals during the prenatal stage of women’s pregnancies, and the relative exclusion of pregnant women from national vaccination projects should be addressed.

## Figures and Tables

**Table 1.** Annual incidence (per 100,000) and numbers of cases of pertussis by age group and calendar year (2001-2018) in Korea

Age (range), yr	2001	2002	2003	2004	2005	2006	2007	2008	2009	2010	2011	2012	2013	2014	2015	2016	2017	2018
Infant (0)	1.403 (8)	2.592 (13)	1.064 (5)	1.086 (5)	2.543 (11)	3.577 (15)	3.129 (14)	1.968 (9)	13.349 (58)	4.582 (20)	10.222 (46)	8.516 (39)	4.516 (20)	9.747 (41)	10.185 (43)	4.644 (19)	9.737 (36)	24.719 (82)
Toddler (1-3)	0.000 (0)	0.108 (2)	0.000 (0)	0.062 (1)	0.000 (0)	0.000 (0)	0.000 (0)	0.000 (0)	0.144 (2)	0.143 (2)	0.503 (7)	0.505 (7)	0.000 (0)	0.423 (6)	1.445 (20)	0.819 (11)	1.301 (17)	4.944 (62)
Preschool (4-6)	0.048 (1)	0.147 (3)	0.000 (0)	0.000 (0)	0.000 (0)	0.057 (1)	0.000 (0)	0.000 (0)	0.000 (0)	0.145 (2)	0.293 (4)	0.000 (0)	0.000 (0)	0.143 (2)	1.581 (22)	0.353 (5)	1.407 (20)	8.215 (114)
School-aged (7-11)	0.000 (0)	0.000 (0)	0.000 (0)	0.000 (0)	0.000 (0)	0.000 (0)	0.000 (0)	0.000 (0)	0.000 (0)	0.035 (1)	0.183 (5)	0.117 (3)	0.083 (2)	0.511 (12)	2.142 (50)	0.996 (23)	4.363 (101)	18.662 (438)
Adolescent (12-19)	0.000 (0)	0.000 (0)	0.000 (0)	0.000 (0)	0.000 (0)	0.000 (0)	0.000 (0)	0.000 (0)	0.054 (3)	0.000 (0)	0.091 (5)	2.895 (157)	0.057 (3)	0.098 (5)	0.490 (24)	0.235 (11)	0.694 (31)	1.646 (70)
Adult (20-59)	0.000 (0)	0.010 (3)	0.000 (0)	0.000 (0)	0.000 (0)	0.003 (1)	0.000 (0)	0.000 (0)	0.010 (3)	0.003 (1)	0.084 (26)	0.071 (22)	0.032 (10)	0.038 (12)	0.093 (29)	0.067 (21)	0.138 (43)	0.233 (72)
Senior young (60-74)	0.000 (0)	0.000 (0)	0.000 (0)	0.000 (0)	0.000 (0)	0.000 (0)	0.000 (0)	0.000 (0)	0.000 (0)	0.018 (1)	0.070 (4)	0.034 (2)	0.016 (1)	0.127 (8)	0.136 (9)	0.289 (20)	0.444 (32)	0.770 (58)
Senior old (≥75)	0.000 (0)	0.000 (0)	0.000 (0)	0.000 (0)	0.000 (0)	0.000 (0)	0.000 (0)	0.000 (0)	0.000 (0)	0.000 (0)	0.000 (0)	0.000 (0)	0.000 (0)	0.080 (2)	0.300 (8)	0.671 (19)	1.251 (38)	2.601 (84)

Values are presented as incidence rates (number of cases).

**Table 2.** Goodness-of-fit test for each model

Model	Deviance (df)	Log likelihood	AIC
Age	5,997.32 (160)	-3,231.19	39.92
Age-period	3,095.69 (159)	-1,780.38	22.02
Age-cohort	3,095.69 (159)	-1,780.38	22.02
Age-period-cohort (intrinsic estimator)	345.65 (100)	-405.36	5.77

Df, degree of freedom; AIC, Akaike information criterion.

## SUPPLEMENTARY MATERIALS

Supplementary Material 1. What is Poisson age-period-cohort (APC) model?
