# Supplementary material for: Recent increase in pertussis incidence in Korea: an age-period-cohort analysis
Source: Epidemiol Health. 2021 Aug 18;43:e2021053. doi: 10.4178/epih.e2021053 (PMC8666685; doi:10.4178/epih.e2021053)
Supplement: Supplementary Material 1. — What is Poisson age-period-cohort (APC) model? [file epih-43-e2021053-suppl.docx]

**Supplementary Material 1. What is Poisson age-period-cohort (APC) model?**

Poisson age-period-cohort (APC) analysis refers APC analysis that the data used for analysis assumed to have a Poisson distribution. Given that almost health data is collected in counted form, APC analysis using health data almost belongs to Poisson APC analysis. Also, we can check whether the given data follows the Poisson distribution using R code (dpois, chisp.test).

Basically, the APC analysis is one of the multiple regression models consisted of age, period, and cohort effect [1]. In here, $Y$ is dependent variable, $\alpha$ is intercept, $\beta{}_{1}$, $\beta{}_{2}$, and $\beta{}_{3}$ are slopes, and $\varepsilon$ is random error [1, 2]. Through the analysis, effect of each variable ($\beta$) is calculated.

$Y=\alpha+ \beta_{1}age+ \beta_{2}period+ \beta_{3}cohort+ \varepsilon$ [1]

However, one of the basic assumption of multiple regression, independency between variables, is not satisfied in APC model due to linear dependency between three variables (e.g., cohort = period-age) [3], so called “identification problem” [1, 4, 5]. To solve the identification problem, many statistical countermeasures have been developed including intrinsic estimator (IE) APC analysis, Hierarchical APC analysis, and Hierarchical APC-Growth Curve model [1].

**Applying IE-APC model**

Among many complements, our study adopted IE-APC model. Intrinsic estimator converts the multiple regression to principal component regression which is free from dependency problem [1, 5]. After conversion, regression coefficients are estimated and the result will be re-converted to original regression model [1, 6, 7]. IE-APC model is preferable tool in health data analysis due to its few restraints. We can obtain robust estimators without any external information or theory, and additional constraint [1, 8].

**Examples**

Application of IE-APC model to infectious disease is well demonstrated in previous researches covered varicella and scarlet fever in children of Jeju-do [9, 10] and hepatitis A [11]. These researches adopted IE-APC analysis and used claims data from the Korean National Health Insurance Corporation [9-11]. As this kind of data take the form of count data, checking whether the data follows Poisson distribution is available.

**References**

1. Heo, J., et al., *The unrealized potential: cohort effects and age-period-cohort analysis.* Epidemiology&health, 2017. **39**.

2. Mason, K.O., et al., *Some Methodological Issues in Cohort Analysis of Archival Data.* American Sociological Review, 1973. **38**(2): p. 242-258.

3. Masters, R. and D. Powers, *Clarifying assumptions in age-period-cohort analyses and validating results.* PLOS ONE, 2020. **15**(10): p. e0238871.

4. Fannon, Z. and B. Nielsen, *Age-Period-Cohort Models*, in *Oxford Research Encyclopedia of Economics and Finance*. 2019.

5. Yang, Y., W.J. Fu, and K.C. Land, *A Methodological Comparison of Age-Period-Cohort Models: The Intrinsic Estimator and Conventional Generalized Linear Models.* Sociological Methodology, 2004. **34**(1): p. 75-110.

6. Yang, Y., *Age-period-cohort analysis : new models, methods, and empirical applications / Yang Yang and Kenneth C. Land*, ed. K.C. Land. 2013, Boca Raton, FL: Boca Raton, FL : CRC Press.

7. Yang Yang, et al., *The Intrinsic Estimator for Age‐Period‐Cohort Analysis: What It Is and How to Use It.* 2008. **113**(6): p. 1697-1736.

8. Land, K.C., et al., *Playing With the Rules and Making Misleading Statements: A Response to Luo, Hodges, Winship, and Powers.* American Journal of Sociology, 2016. **122**(3): p. 962-973.

9. Kim, J., J.-E. Kim, and J.-M. Bae, *Incidence of varicella in children in Jeju-do, Korea, 2005-2016: age-period-cohort analysis.* Epidemiol Health, 2018. **40**(0): p. e2018054-0.

10. Kim, J., J.-E. Kim, and J.-M. Bae, *Incidence of Scarlet Fever in Children in Jeju Province, Korea, 2002-2016: An Age-period-cohort Analysis.* J Prev Med Public Health, 2019. **52**(3): p. 188-194.

11. Seo, J.Y., et al., *Age-period-cohort analysis of hepatitis A incidence rates in Korea from 2002 to 2012.* Epidemiol Health, 2016. **38**(0): p. e2016040-0.
